# Quasi-steady-state air plasma channel produced by a femtosecond laser pulse sequence

**DOI:** 10.1038/srep15515

**Published:** 2015-10-23

**Authors:** Xin Lu, Shi-You Chen, Jing-Long Ma, Lei Hou, Guo-Qian Liao, Jin-Guang Wang, Yu-Jing Han, Xiao-Long Liu, Hao Teng, Hai-Nian Han, Yu-Tong Li, Li-Ming Chen, Zhi-Yi Wei, Jie Zhang

**Affiliations:** 1 Beijing National Laboratory for Condensed Matter Physics, Institute of Physics, Chinese Academy of Sciences, Beijing 100190, China; 2 Key Laboratory for Laser Plasmas (Ministry of Education) and Department of Physics, Shanghai Jiao Tong University, Shanghai 200240, China; 3 IFSA Collaborative Innovation Center, Shanghai Jiao Tong University, Shanghai 200240, China; 4 Academy of Opto-Electronics, Chinese academy of sciences, Beijing, 100094, China; 5 Department of Physics, Heze University, Heze, 274015, China

## Abstract

A long air plasma channel can be formed by filamentation of intense femtosecond laser pulses. However, the lifetime of the plasma channel produced by a single femtosecond laser pulse is too short (only a few nanoseconds) for many potential applications based on the conductivity of the plasma channel. Therefore, prolonging the lifetime of the plasma channel is one of the key challenges in the research of femtosecond laser filamentation. In this study, a unique femtosecond laser source was developed to produce a high-quality femtosecond laser pulse sequence with an interval of 2.9 ns and a uniformly distributed single-pulse energy. The metre scale quasi-steady-state plasma channel with a 60–80 ns lifetime was formed by such pulse sequences in air. The simulation study for filamentation of dual femtosecond pulses indicated that the plasma channel left by the previous pulse was weakly affected the filamentation of the next pulse in sequence under our experimental conditions.

The plasma filament (also called the plasma channel) generated by femtosecond laser pulses in air has been studied intensively over the past two decades^1^,^2^,^3^,^4^,^5^,^6^,^7^,^8^ because of a series of attractive applications, such as remote sensing^3^,^9^,^10^, lightning control^11^,^12^,^13^,^14^,^15^,^16^,^17^,^18^,^19^,^20^, terahertz generation^21^,^22^, guiding of radiofrequency energy^23^,^24^, and even triggering rain or snow^25^,^26^. Many experimental studies have shown that the lifetime of a plasma channel produced by a single femtosecond laser pulse is only several nanoseconds due to the recombination process^27^. This causes difficulties for large-scale applications based on the conductivity of the plasma channel. A promising approach to prolonging the channel lifetime is to heat the electron plasma by a long pulse via inverse Bremsstrahlung. This method has been investigated and practiced in a filament-triggered HV discharge experiment^13^,^19^,^28^. However, long pulses cannot support the filament over long distance because long pulses are difficult to propagate over long distances without diffraction loss. Another way to produce a plasma channel with a long lifetime is by using an ultra-short laser pulse sequence to produce temporally separated but spatially overlapping filaments. With this method, the lifetime of the filament was doubled by using dual femtosecond laser pulses^29^. Ji *et al.* used the “leaking” pulses from a regenerative amplifier as the multi-pulse seed and produced a sequence of approximately 6 pulses, prolonging the lifetime of the plasma channel by a factor of 4.5 (see ref. 30). Later, Ionin *et al.* generated a train of picosecond ultraviolet (UV) pulses overlapped with a long UV pulse by a hybrid Ti:sapphire-KrF laser facility, where the pulse train seed was obtained by multi-reflection of a UV femtosecond laser pulse in a ring cavity^31^,^32^,^33^. The femtosecond pulse sequence at an 800 nm central wavelength was also produced by pure multi-pass amplification of the “natural” pulse sequence from a 70 MHz commercial femtosecond oscillator^34^. However, the interval between pulses (14 ns) was still much longer than the channel lifetime, and the uniformity of the pulse energy in sequence was rather poor. Under those conditions, a long-lifetime plasma channel could be generated only by the tight focusing of such laser pulse sequences in air, and the length of the plasma channel was only several mm. When the pulse sequence was weakly focused in air, only approximately 3 of the strongest pulses in sequence could efficiently generate the plasma channel due to the poor uniformity, and the plasma channel was periodically interrupted due to long intervals between the pulses.

To achieve a breakthrough in the quality of femtosecond pulse sequence, the interval between pulses needs to be reduced to a few nanoseconds, and the number of amplified pulses needs to be increased further. In this study, we used the output from a home-made 350 MHz repetition rate (corresponding only to a 2.9 ns interval) femtosecond oscillator as the seed and successfully generated an amplified pulse sequence with more than 20 pulses in 70 mJ total energy. The lifetime of the plasma channel generated by such a pulse sequence was prolonged up to 60–80 ns (bottom width), while the length of the filament was simultaneously kept in metre scale. Moreover, the coupling between pulses in sequence during filamentation was studied by numerical simulations.

## Results

**Figure 1:**
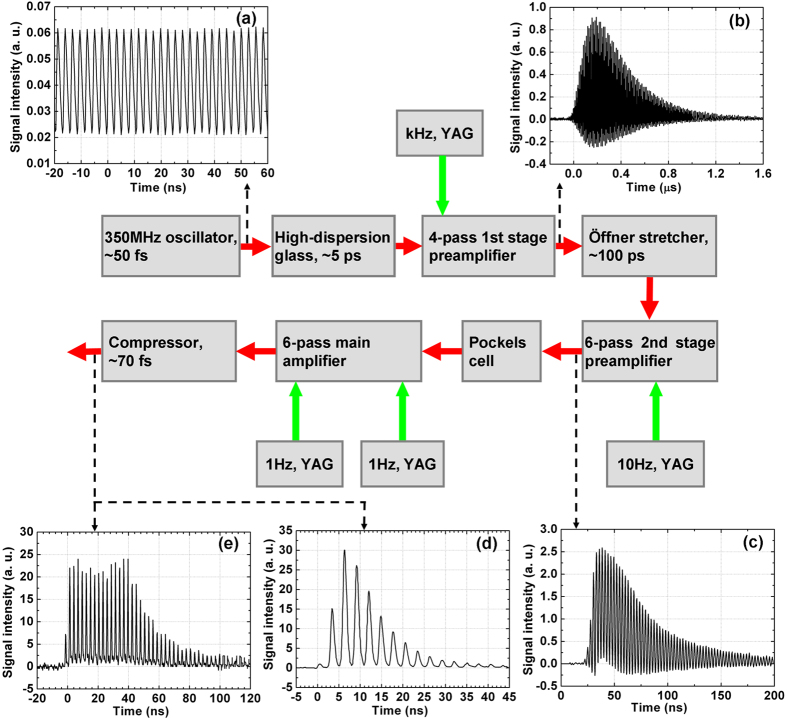
The scheme of the laser system for the generation of a femtosecond pulse sequence and the photodiode signals of the pulse sequence at different stages. (**a**) From oscillator, (**b**) after 1st preamplifier, (**c**) after 2nd preamplifier, (**d**) final output optimized for maximum total energy, and (**e**) final output optimized for best uniformity.

The experiments were carried out using a modified Ti:sapphire CPA laser system, which uses a pure multi-pass amplification chain, as shown in Fig. 1. The seed pulse sequence (the photodiode signal of which is presented in Fig. 1a) from a home-made 350 MHz, 50 fs oscillator was first stretched by a high-dispersion glass to a few picosecond-pulse duration and then amplified by a 4-pass 1st-stage preamplifier without selection. After the 1st preamplification, a pulse sequence with a width of approximately 400 ns (FWHM) and ~0.2 mJ total energy was obtained. The photodiode signal of the pulse sequence after the 1st-stage preamplifier is shown in Fig. 1b. Then, the pulses in the sequence were stretched to ~100 ps by an Öffner stretcher and amplified by a 6-pass 2nd stage preamplifier. After the 2nd preamplification, the width (FWHM) of the pulse sequence was reduced to 50 ns (Fig. 1c). The total energy of the pulse sequence was increased to ~3 mJ. Finally, the pulses in sequence were amplified by a six-pass main amplifier and then compressed to a duration of ~70 fs. The main amplifier was pumped by two YAG lasers at a 1 Hz repetition rate and 700 mJ pulse energy. When two synchronous pump pulses pumped the Ti:sapphire crystal, we achieved an output pulse sequence with maximum total energy of 120 mJ (Fig. 1d). It can be observed that fewer than ten pulses are efficiently amplified, and the uniformity of the pulses was very poor. The reason is that pulses in the front edge of the sequence extract much more energy from the Ti:sapphire crystal during each pass. We have successfully suppressed the gain competition between pulses by introducing some delay between the two pump pulses of the main amplifier. The pulse sequence with optimized uniformity was obtained under the condition of a 50 ns delay between the two pump pulses. Figure 1e shows the photodiode signal of the pulse sequence optimized for best uniformity, but the cost is that the total energy of the pulse sequence was reduced to approximately 70 mJ. Figure 1e shows a flat top that contains more than ten pulses. The pulses in the flat-top area correspond to approximately 3 mJ energy and 43 GW peak power. It should be noted that the energy distribution of pulses in sequence undergoes some fluctuations from shot to shot, but the overall distribution of the pulse sequence is much better than the regime of maximum total energy, as shown in Fig. 1d.

**Figure 2:**
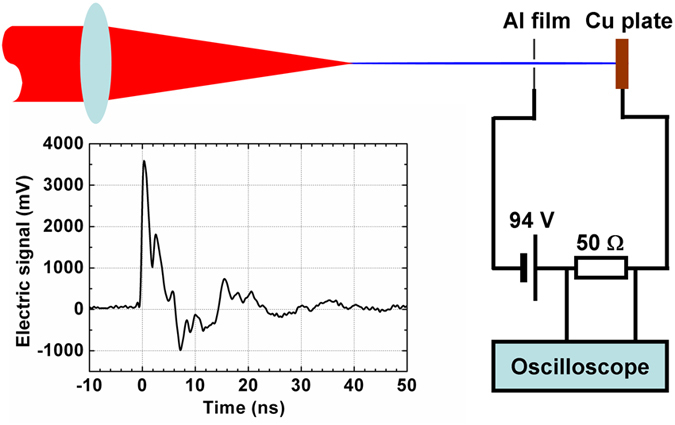
Setup of electric diagnostics of the plasma channel and an example of the electric signal of the plasma channel generated by a single femtosecond laser pulse.

The experiment was performed using a pulse sequence optimized for best uniformity, as shown in Fig. 1e. The pulse sequence was focused by lenses of different focal lengths, *f *=2 m and *f *= 4 m, to generate the plasma channel in air. The properties of the plasma channel were detected by electric conductivity measurement, which is frequently used for diagnostics of plasma channels^27^,^35^,^36^,^37^,^38^,^39^. The setup used in our experiment is shown in Fig. 2.

A direct current voltage of 94 V was applied to two plane electrodes through a 50 Ω divider resister. The distance between the two electrodes was 15 mm. The plasma channel connected two electrodes and triggered transient current in the circuit, and the voltage signal on the 50 Ω resister was recorded by the oscilloscope. To reduce the contact resistance between the plasma channel and the electrodes, the electrode to the side of the laser pulses was a thin (15 μm) aluminium film, and the other electrode was a thick copper plate. The plasma channel first burned a hole with a diameter of approximately 0.5 mm on the film electrode by several shots, and then passed through it to the thick copper electrode. Before the experiment with pulse sequences, we tested our electric measurement device on a single-pulse plasma channel, which was generated by focusing a 11 mJ, 30 fs laser pulse from another laser system by a *f *= 1 m convex lens in air. The voltage signal recorded on the strongest part of such a plasma channel is shown in Fig. 2 and is very similar to the electric signal obtained in ref. 35, which used almost the same diagnostic setup. The rise time of the voltage signal was only 0.8 ns, and the FWHM of the signal was 1.6 ns. The small fluctuation of signal after the main peak was induced by the self-oscillation of the circuit. This result indicates that the lifetime of the plasma channel produced by a single femtosecond pulse is only a few ns, which is in good agreement with the earlier diagnostics using electric conductivity measurement^27^,^35^, time-resolved fluorescence measurement^29^, and a microwave probe^40^.

**Figure 3:**
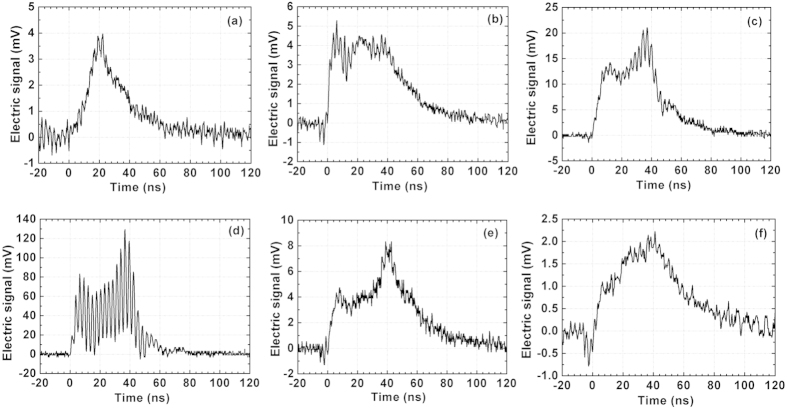
Electric signal of the plasma channel under *f* =* *2 m external focusing at different distances. (**a**) *z *= 0 cm, (**b**) *z *= 5 cm, (**c**) *z *= 10 cm, (**d**) *z *= 20 cm, (**e**) *z *= 25 cm, and (**f**) *z *= 35 cm.

The pulse sequence was focused into air by convex lenses with different focal lengths—2 m and 4 m—forming plasma channels with lengths of 35 cm and 1.1 m, respectively. The electric signal of the plasma channel was measured along the propagation distance by a step of 2.5 cm. Figure 3 shows some typical electric signals of plasma channels generated using the *f* = 2 m convex lens at different positions on the plasma channel: z = 0 cm, 5 cm, 10 cm, 20 cm, 25 cm, and 35 cm. The zero distance corresponds to the start point of plasma channel, where the electric signal can barely be detected. It can be observed that the peak signal intensity varied from several mV to more than 100 mV for different propagation distances, but the temporal width of the signal always remained in the 80 ns range (bottom width) due to the improved uniformity of the pulse sequence.

**Figure 4:**
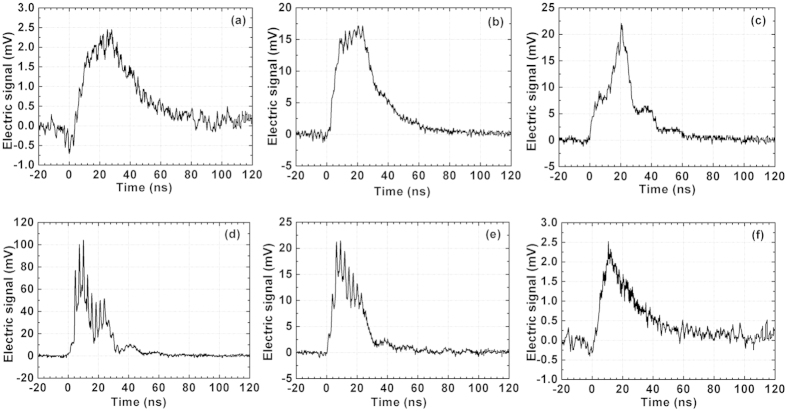
Electric signal of the plasma channel under *f *=* *4 m external focusing at different distances. (**a**) *z *= 0 cm, (**b**) *z *= 25 cm, (**c**) *z *= 37.5 cm, (**d**) *z *= 60 cm, (**e**) *z *= 85 cm, and (**f**) *z *= 112.5 cm.

The electric signals of the plasma channel under external focusing of *f *= 4 m are shown in Fig. 4 for distances of 0, 25 cm, 37.5 cm, 60 cm, 85 cm and 112.5 cm. The lifetime of the channel with *f *= 4 m external focusing was reduced to approximately 60 ns because under this condition only about ten of the strongest pulses in sequence can efficiently form plasma channels.

**Figure 5:**
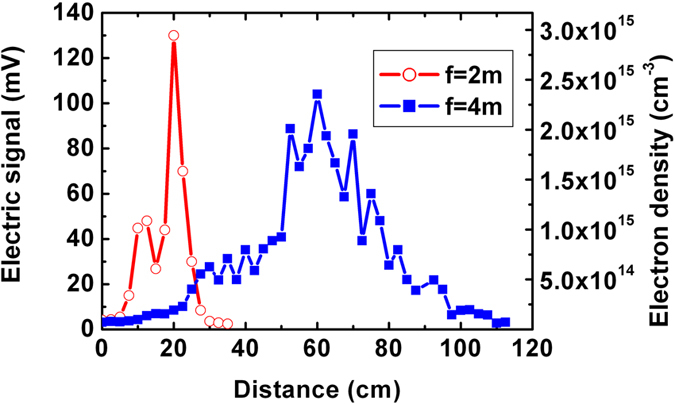
Peak electric signal (left vertical axis) and electron density (right vertical axis) of the plasma channel under initial focusing conditions *f *= 2 m (open circle) and *f *=* *4 m (square).

The resistance of the plasma channel between the two electrodes can be calculated from the electric signal by Ohm’s law. Furthermore, we can simply estimate the electron density of the plasma channel by the formula for electrical conductivity of plasma^37^:


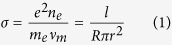


where *e* is the elementary charge; *n*_*e*_ is the electron density; *m*_*e*_ is the electron mass; 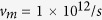 is the collisional frequency for electrons in laboratory conditions^37^; and *R*, *l*, and *r* are the resistance, length and radius of the plasma column, respectively. The radius of the plasma channel is estimated to be 40 μm from an earlier experiment with similar peak power and numerical aperture, described in ref. 41. Therefore, the electron density can be easily calculated according to formula (1). Figure 5 shows the peak electric signal intensity (left vertical axis) and electron density (right vertical axis) along the plasma channel for different external focuses of *f* = 2 m and 4 m. The zero distance corresponds to the start position of each channel. We also tried to measure the contact resistance between the plasma channel and the film electrode by linear extrapolation of the dependencies between the resistance and the length of the plasma channel, but the results indicated that the contact resistance was at noise level and can be ignored.

For a deep understanding of the filamentation mechanism of a femtosecond pulse sequence, the effect of plasma produced by the previous pulse on the filamentation of the next pulse in sequence should be considered. We have performed numerical simulations on the filamentation of two identical femtosecond pulses with a 2.9 ns time delay to study the coupling between two consecutive pulses in sequence. The energy, initial diameter (FWHM) and duration of each pulse were set at 3 mJ, 10 mm and 70 fs, respectively, as our experimental conditions. The geometric focal length of the dual pulses was set at 2 m. However, due to the limitations of computational resources, the initial profile of the laser beam was scaled to a 1 mm diameter and 200 mm focal length in simulation to achieve enough high-spatial resolution. The simulation of dual-pulse filamentation was performed in 3 steps: first, we simulated the filamentation of the first pulse and obtained the spatial distribution of electron density; second, we calculated the time evolution of electron density during the free decay period and obtained the electron density field just before the arrival of the second pulse; and third, we simulated the filamentation of the second pulse in the decayed electron density field left by the first pulse. The electron diffusion can be ignored because it is a slow process. The ionization from exited states of air molecules can also be ignored during preliminary study.

The filamentation of the first pulse was modelled by numerically solving the 3D + 1 nonlinear Schrödinger equation (NLS) with higher-order Kerr terms^42^,^43^,^44^. The free electrons were generated mainly by multi-photon ionization of oxygen molecules in the air. The coupled equations can be written as


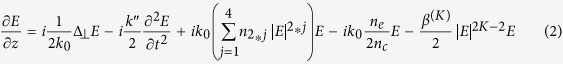



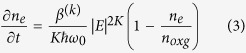


These equations are expressed in the reference frame moving with the pulse group velocity (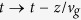) where *E* is the complex amplitude of the laser pulse envelope and 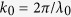 is the central wave number. The terms on the right-hand side of Eq. (2) account for the transverse diffraction with 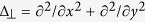; the group velocity dispersion; the Kerr nonlinearity, including higher-order effect; the defocusing effect induced by plasma; and the power dissipation caused by multi-photon absorption with the following coefficients: 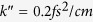; 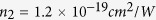, 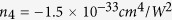, 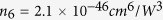, and 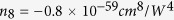
(see ref. 44); and 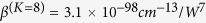
(see ref. 45), where 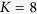 is the number of photons needed to extract electrons from neutral oxygen molecules at an 800 nm wavelength. 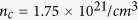
and 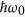 are the critical plasma density and single-photon energy at the central laser wavelength, respectively. The density of neutral oxygen molecules in air is 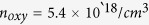.

**Figure 6:**
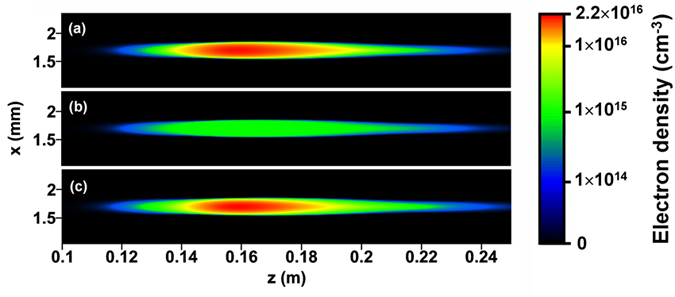
The electron density distribution of the plasma channel at different points in time: (**a**) after the first pulse; (**b**) just before the second pulse; and (**c**) after the second pulse.

Equations (2) and (3) were solved using full-step (not split-step) Fast Fourier Transformation in transverse space and time dimensions with 13 μm spatial resolution and 1.9 fs time resolution. The initial profile of the laser pulse was set to be Gaussian shape in time and space. Figure 6a shows the electron density distribution of the plasma channel formed by the first pulse. The maximum electron density is 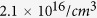, which is in good agreement with the simulation result in ref. 46 under similar laser peak power and numerical aperture. The ideal symmetry and smoothness of the initial beam profile used in simulation made the electron density of the plasma channel higher than the experimental results; this situation also occurred in ref. 46, but it does not affect the consideration of physical mechanisms.

**Figure 7:**
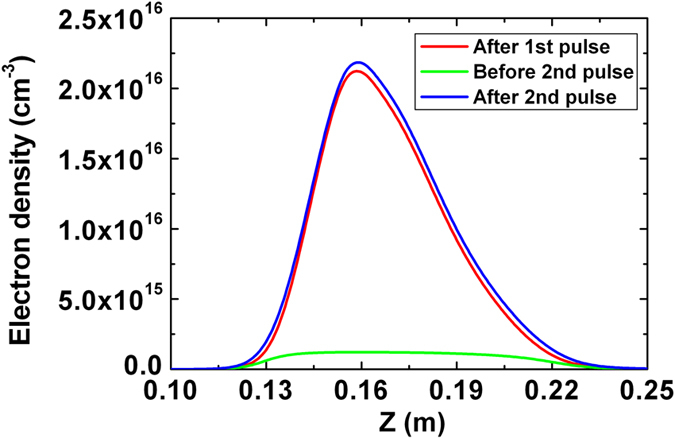
On-axis electron density of the plasma channel at different points in time: red line – after the 1st pulse, green line – before the 2nd pulse, and blue line – after the 2nd pulse.

The time evolution of electron density in the free decay period is described by the following formula^27^:


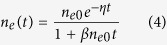


where *n*_*e*0_ is the initial electron density. In laboratory conditions, 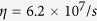 is the attachment coefficient, and 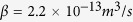 is the recombination coefficient^13^. After a 2.9 ns free decay period, the electron density field felt by the first pulse is shown in Fig. 6b. The calculation by formula (4) shows that the decay rate of the plasma channel is strongly dependent on the initial electron density. The maximum electron density decayed from 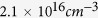 to 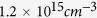. In the weak part of the channel, where the initial electron density was 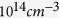 (blue area in Fig. 6), the reduction of electron density was only 22%. The filamentation of the second pulse can also be described by equations (2) and (3), but with different initial conditions for electron density:


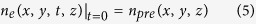


where 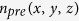 is the electron density field left by the first pulse, as shown in Fig. 6b. The electron density of the plasma channel after the second pulse is shown in Fig. 6c. Figure 7 shows the on-axis electron density of the plasma channel at three different points in time: after the first pulse, just before the second pulse, and after the second pulse. It can be observed in Figs 6 and 7 that the distributions of electron density produced by first and second pulses are almost the same, and the final electron density was contributed primarily by the second pulse. These results indicate that the defocusing effect of electron density left by the first pulse on the propagation of the second pulse is rather weak. The two pulses formed plasma channels largely independently.

## Discussion

In our experiment, the maximum electron density of the plasma channel was much lower than that of the simulation results; therefore, the coupling between pulses in sequence can be ignored. Close to the start and end positions of the plasma channel, the electron density produced by each pulse in sequence is very low, at only 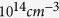. In the free period between pulses, the weak parts of the plasma channel decayed very slowly, and therefore the electron density accumulated with the number of pulses. On the other hand, when the electron density increased, the recombination process also became faster. When the recombination during the free decay period reached balance with the ionization by the next pulse, the electron density was stabilized, and we can see a plateau area in the time evolution of the electric signal, as shown in Fig. 3b,c. In the trailing edge of the pulse sequence, when the ionization could not compensate for the recombination, the electron density decreased with the envelope of pulse sequences. In the middle part of the channel, the electron density produced by each pulse is 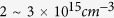, and ~70% electron density was decayed in the period between two consecutive pulses. This factor led to a strong oscillation of electric signals within the same period as the pulse sequence. Sometimes, the self-oscillation in the circuit dropped the electric signal down to a negative value, as shown in Fig. 4(d), but this factor would not affect the features of the plasma channel. It should be noted that even very small variations in the pulse beam profile or intensity can alter the position of the filaments. Further improvements in plasma channel quality will be challenged to confine the individual filaments in one spatial location. In our opinion, the point stability of each individual filament could be enhanced by improving the beam quality and radial symmetry of the beam profile.

In conclusion, we have developed a new laser source to produce 350 MHz femtosecond pulse sequences with greatly enhanced pulse numbers and uniformity. The metre scale quasi-steady-state plasma channel with a 60–80 ns lifetime was produced by such a pulse sequence. The main mechanism of such long-lifetime plasma channel is the periodic creation of free electrons via consecutive photo-ionization. The heating of the plasma seems to be negligible due to low electron density and short laser pulse duration. Numerical simulations for the filamentation of dual femtosecond pulses with a 2.9 ns time interval indicated that the coupling between pulses during filamentation was rather weak, and each laser pulse in sequence formed the plasma channel almost independently. Currently, the output energy of femtosecond laser systems can reach joules and even tens of joules, which can support sequencing of hundreds of pulses with a hundred mJ level single-pulse energy. Therefore, we think there is no principle limitation to generate kilometre-range plasma channels with microsecond-level lifetimes, which will be useful for many applications such as lightning control and remote transportation of radio frequency energy. Moreover, as a new type of femtosecond laser source, the femtosecond laser pulse sequence can bring new advantages to remote sensing, terahertz generation, and femtosecond laser fabrications. As a new type of filamentation, there are still many unknown fundamental physical problems that need to be studied further.

## Methods

The numerical scheme runs along axis *z* by step 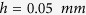, and the numbers of numerical grids *x*, *y*, and *t* were 256, 256 and 128, respectively. The complex amplitude 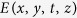 was first transformed into a frequency domain 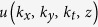 by 3-Dimensional Finite-Fourier-Transformation. Then, the NLS equation (2) could be transformed to a set of ordinary differential equations for 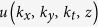. The laser field on the next layer, 
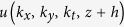
, was obtained by a finite difference scheme for 
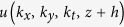
 and 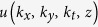. The nonlinear terms were calculated by an iterative procedure that is started by replacing 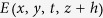 with 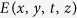. Normally, the iteration converged after 6–7 cycles. Unlike in the split-step Fourier method, in our simulation, the linear and nonlinear operators were considered simultaneously during each iteration. We also tested the stability of our numerical scheme by using half the number of spatial grids, but the difference in results was quite small and can be ignored.

## Additional Information

**How to cite this article**: Lu, X. *et al.* Quasi-steady-state air plasma channel produced by a femtosecond laser pulse sequence. *Sci. Rep.* **5**, 15515; doi: 10.1038/srep15515 (2015).
